# Apamin Does Not Inhibit Human Cardiac Na^+^ Current, L-type Ca^2+^ Current or Other Major K^+^ Currents

**DOI:** 10.1371/journal.pone.0096691

**Published:** 2014-05-05

**Authors:** Chih-Chieh Yu, Tomohiko Ai, James N. Weiss, Peng-Sheng Chen

**Affiliations:** 1 Krannert Institute of Cardiology and Division of Cardiology, Department of Medicine, Indiana University School of Medicine, Indianapolis, Indiana, United States of America; 2 Department of Integrated Diagnostic & Therapeutics, National Taiwan University, Taipei, Taiwan; 3 Department of Molecular Pathogenesis, Division of Pathophysiology, Medical Research Institute, Tokyo Medical and Dental University, Tokyo, Japan; 4 Cardiovascular Research Laboratory, Departments of Medicine (Cardiology) and Physiology, David Geffen School of Medicine, University of California Los Angeles, Los Angeles, California, United States of America; Dalhousie University, Canada

## Abstract

**Background:**

Apamin is commonly used as a small-conductance Ca^2+^-activated K^+^ (SK) current inhibitor. However, the specificity of apamin in cardiac tissues remains unclear.

**Objective:**

To test the hypothesis that apamin does not inhibit any major cardiac ion currents.

**Methods:**

We studied human embryonic kidney (HEK) 293 cells that expressed human voltage-gated Na^+^, K^+^ and Ca^2+^ currents and isolated rabbit ventricular myocytes. Whole-cell patch clamp techniques were used to determine ionic current densities before and after apamin administration.

**Results:**

Ca^2+^ currents (CACNA1c+CACNB2b) were not affected by apamin (500 nM) (data are presented as median [25^th^ percentile;75^th^ percentile] (from –16 [–20;–10] to –17 [–19;–13] pA/pF, P = NS), but were reduced by nifedipine to –1.6 [–3.2;–1.3] pA/pF (p = 0.008). Na^+^ currents (SCN5A) were not affected by apamin (from –261 [–282;–145] to –268 [–379;–132] pA/pF, P = NS), but were reduced by flecainide to –57 [–70;–47] pA/pF (p = 0.018). None of the major K^+^ currents (*I*_Ks_, *I*_Kr_, *I*_K1_ and *I*_to_) were inhibited by 500 nM of apamin (KCNQ1+KCNE1, from 28 [20]; [37] to 23 [18]; [32] pA/pF; KCNH2+KCNE2, from 28 [24]; [30] to 27 [24]; [29] pA/pF; KCNJ2, from –46 [–48;–40] to –46 [–51;–35] pA/pF; KCND3, from 608 [505;748] to 606 [454;684]). Apamin did not inhibit the *I*_Na_ or *I*_CaL_ in isolated rabbit ventricular myocytes (*I*_Na,_ from –67 [–75;–59] to –68 [–71;–59] pA/pF; *I*_CaL_, from –16 [–17;–14] to –14 [–15;–13] pA/pF, P = NS for both).

**Conclusions:**

Apamin does not inhibit human cardiac Na^+^ currents, L-type Ca^2+^ currents or other major K^+^ currents. These findings indicate that apamin is a specific SK current inhibitor in hearts as well as in other organs.

## Introduction

Small-conductance calcium activated potassium (SK) channels, which are abundantly present in the central nervous system [1], were first cloned in 1996 by Kohler *et al* [2]. Study of this channel is facilitated by the use of apamin, which has been thought to be a specific inhibitor of SK current in the nervous system [1], [3], [4]. Subsequent investigations showed that the apamin-sensitive potassium current (*I*_KAS_) is present in the atria [5]–[12]. In addition, while normal ventricles paced at physiological cycle lengths do not express significant *I*_KAS_ [13], we and others found that *I*_KAS_ expression is upregulated in failing, ischemic or infarcted human, rabbit and rat ventricles and in normal rabbit ventricles with complete atrioventricular block [14]–[19]. A common criticism of all these studies is that the specificity of apamin in cardiac type ion channels has not been well established. Some previous studies have shown that apamin inhibits fetal L-type Ca^2+^ currents [20]–[22] and Na^+^ currents [23] in the chick heart, suggesting that apamin may have off target effects on other cardiac ion channels. However, there is no information on the effects of apamin on Na^+^, Ca^2+^ and K^+^ currents that are responsible for adult human cardiac activation and repolarization. Because *I*_KAS_ is potentially important in human cardiac arrhythmogenesis, it is important to establish whether apamin is a specific SK current inhibitor as apamin is used to define *I*_KAS_. The purpose of the present study was to test the hypothesis that apamin is a specific inhibitor of *I*_KAS_ in adult human cardiac ion channels. We tested that hypothesis by performing patch clamp studies of major cardiac ion channels expressed in human embryonic kidney (HEK) 293 cells and by testing the effects of apamin on Na^+^ and Ca^2+^ currents in rabbit ventricular myocytes.

## Materials and Methods

The study was approved by the Institutional Biosafety Committee and Institutional Animal Care and Use Committee of the Indiana University and the Methodist Research Institute, Indianapolis, Indiana.

### Cell Culture and Gene Transfection

Human embryonic kidney (HEK) 293 cells were cultured in Iscove’s Modified Dulbecco’s Medium (Gibco) with 10% fetal bovine serum and 1% penicillin/streptomycin in 5% CO_2_ at 37°C. To study human Nav1.5, a stable HEK 293 cell line expressing consistent sodium currents (*I*_Na_) was used [24]. Other than *I*_Na_, 35 mm dishes of HEK 293 cells were transiently transfected using Effectene Transfection Reagent (Qiagen) according to the manufacturer’s protocol and were harvested for patch clamp experiment 48∼72 hours later. The amount and content of plasmids transfected for each channel were described as followings: for *I*_Ca_, 1.5 µg of CACNA1c/pcDNA3.1 and 2.0 µg of CACNB2b/pIRES2-DsRed-Express were co-transfected; for *I*_Ks_, 1 µg of KCNQ1/pIRES2-EGFP and 1 µg of KCNE1/pIRES-CD8 were co-transfected; for *I*_Kr_. 3 µg of KCNH2/pIRES-hyg and 1 µg of KCNE2/pIRES2-DsRed-Express were co-transfected; for *I*_K1_, 2 µg of KCNJ2/pCMS-EGFP were transfected; and for *I*_to_, 2 µg of KCND3/pIRES2-DsRed-Express were transfected.

The stably SK2-expressing cells were used for positive control studies to test the effects of apamin. The SK2 clone was developed in our laboratory. HEK 293 cells were transfected with 2.0 µg of KCNN2/pIRES-hyg plasmids. Single cells were picked and propagated in selection media containing hygromycin 200 µg/ml. Expression of *I*_SK2_ was verified by patch-clamp measurements.

### Rabbit Cardiomyocyte Isolation

The rabbits were intravenously injected with 1,000 units of heparin and anesthetized with sodium pentobarbital (100 mg/kg). After a median sternotomy, the hearts were rapidly excised, mounted onto a Langendorff perfusion apparatus and perfused for 4 minutes with 37°C oxygenated Ca^2+^-free buffer. The composition of the buffer was (in mM) NaCl 136, KCl 5.4, NaH_2_PO_4_ 0.33, MgCl_2_ 1.0, HEPES 10 and glucose 10, adjusted to pH 7.4 with NaOH. After the blood was washed out, the heart was recirculated with enzyme solution, containing 1 mg/ml collagenase type II (Worthington, Lakewood, NJ) and 0.1 mg/ml protease (Sigma-Aldrich, St. Louis, MO, USA) in the same buffer for 28 minutes, followed by another 4 minutes of washing with Ca^2+^-free buffer. The heart was then removed from the apparatus and the ventricle was triturated. The isolated cardiomyocytes were washed and titrated up with Ca^2+^-containing Tyrode’s solution until the Ca^2+^ level reaches 1.8 mM.

### Patch-clamp Experiments

Whole cell configuration of the voltage-clamp technique was used in this study as described elsewhere [25]. Briefly, whole-cell configuration was made in Tyrode’s solution. Pipette resistances were 1.5–3 MΩ. After achieving a gigaseal, the test-pulse current was nulled by adjusting the pipette capacitance compensator with both fast and slow components. After break-in, the whole-cell charging transient was nulled by adjusting whole cell capacitance and series resistance. Voltage control protocols were generated with Axopatch 200B amplifier/Digidata 1440A acquisition system using pCLAMP-10 software (Molecular Devices/Axon, Sunnyvale, CA). Whole-cell recording was analyzed using Clampfit 10.2. To measure *I*_SK2_, we used Tyrode’s solution as the bath solution containing (in mM) NaCl 140, KCl 5.4, MgCl_2_ 1.2, HEPES 5, NaH_2_PO_4_ 0.33, CaCl_2_ 1.8 and Glucose 10 (pH 7.4 adjusted with NaOH). The pipette solution contained (in mM) K-Gluconate 144, MgCl_2_ 1.15, EGTA 1, HEPES 10 and free Ca^2+^1 µM (pH 7.2 adjusted with KOH). All experiments for *I*_SK2_ were carried out at 37°C. For measuring *I*_Na_, we used Tyrode’s solution (see above) as the bath solution. The pipette solution contained (in mM) NaF 10, CsF 110, CsCl 20, EGTA 10, and HEPES 10 (pH 7.35 adjusted with CsOH). After testing apamin 500 nM, fleicainide 100 µM was used as a positive control [26]. For measuring *I*_Ca_, we replaced extracellular calcium with barium to lessen the rundown phenomenon [27], [28]. The bath solution contained (in mM) BaCl_2_ 5, NaCl 130, MgCl_2_ 1.0, HEPES 10, and Glucose 11 (pH 7.4 adjusted with NaOH). The pipette solution contained (in mM) CsCl 120, MgCl_2_ 2, EGTA 10, HEPES 10, Mg-ATP 5, Na_2_-GTP 1.5 and cAMP 1 (pH 7.24 adjusted with CsOH). Nifedipine 2 µM was used as the positive control [29]. All experiments for *I*_Ba_ were carried out at 37°C. For measuring *I*_Ks_, we used Tyrode’s solution as the bath solution (see above). The pipette solution contained (in mM) KCl 130, KOH 20, EGTA 5, Mg-ATP 5, HEPES 5, cAMP 0.05 and Na_2_-GTP 0.1 (pH 7.4 adjusted with KOH). Chromanol 293B 50 µM was used as the positive control [30]. For measuring *I*_Kr_, *I*_K1_ and *I*_to_, we used Tyrode’s solution (see above) as the bath solution. The pipette solution contained (in mM) KCl 130, KOH 20, EGTA 5, Mg-ATP 5 and HEPES 5 (pH 7.4 adjusted with KOH). E4031 100 nM, CsCl 5 mM and 4-aminopyridine 10 mM were used as the positive control, respectively [31]–[33]. To measure *I*_Na_ and *I*_Ca_ in rabbit cardiomyocytes, we used Tyrode’s solution as the bath solution (see above), and the pipette solution contained (in mM) aspartate 85, TEACl 20, MgCl_2_ 2, EGTA 10, HEPES 10, Mg-ATP 5, and Na_2_-GTP 5 (pH 7.2 adjusted with KOH).

Stable current density during baseline solution superperfusion was measured immediately before the addition of apamin to define baseline current density. This was followed by superfusion with 500 nM apamin for at least 3 minutes until the current became stable. Following apamin exposure, specific blockers of each current were used as positive controls.

### Drugs and Reagents

Apamin (catalog#1652), was purchased from Tocris Bioscience (Minneapolis, MN) and was dissolved in water for a 250 µM stock solution. Apamin was freshly diluted with bath solution daily before experiment. Flecainide (catalog#1470), chromanol 293B (catalog#1412) and E4031 (catalog#1808) were purchased from Tocris. All other chemicals were purchased from Sigma-Aldrich (St. Louis, MO).

### Statistical Analysis

Summary data following apamin or positive controls were normalized to baseline. Nonparametric tests were used in this whole experiment. Related-samples Friedman’s Two-Way Analysis of Variance by Ranks was conducted to compare continuous variables among baseline, post apamin and post specific blockers. Related-Samples Wilcoxon Signed Rank Test was performed for post-hoc analysis. *I*_Ks_ rundown was quantified by the time constant (τ) of a single exponential fit of the current. Independent-samples Mann-Whitney U test was performed to compare τ of rundown with and without apamin. P value less than 0.05 was considered statistically significant. Statistical analyses were performed using SPSS (IBM, Chicago, IL, USA, version 21). Data in text and figures are presented as median [25^th^ percentile;75^th^ percentile].

## Results

### Studies in HEK 293 Cells

#### Apamin’s effects on I_SK2_

**Figure 1:**
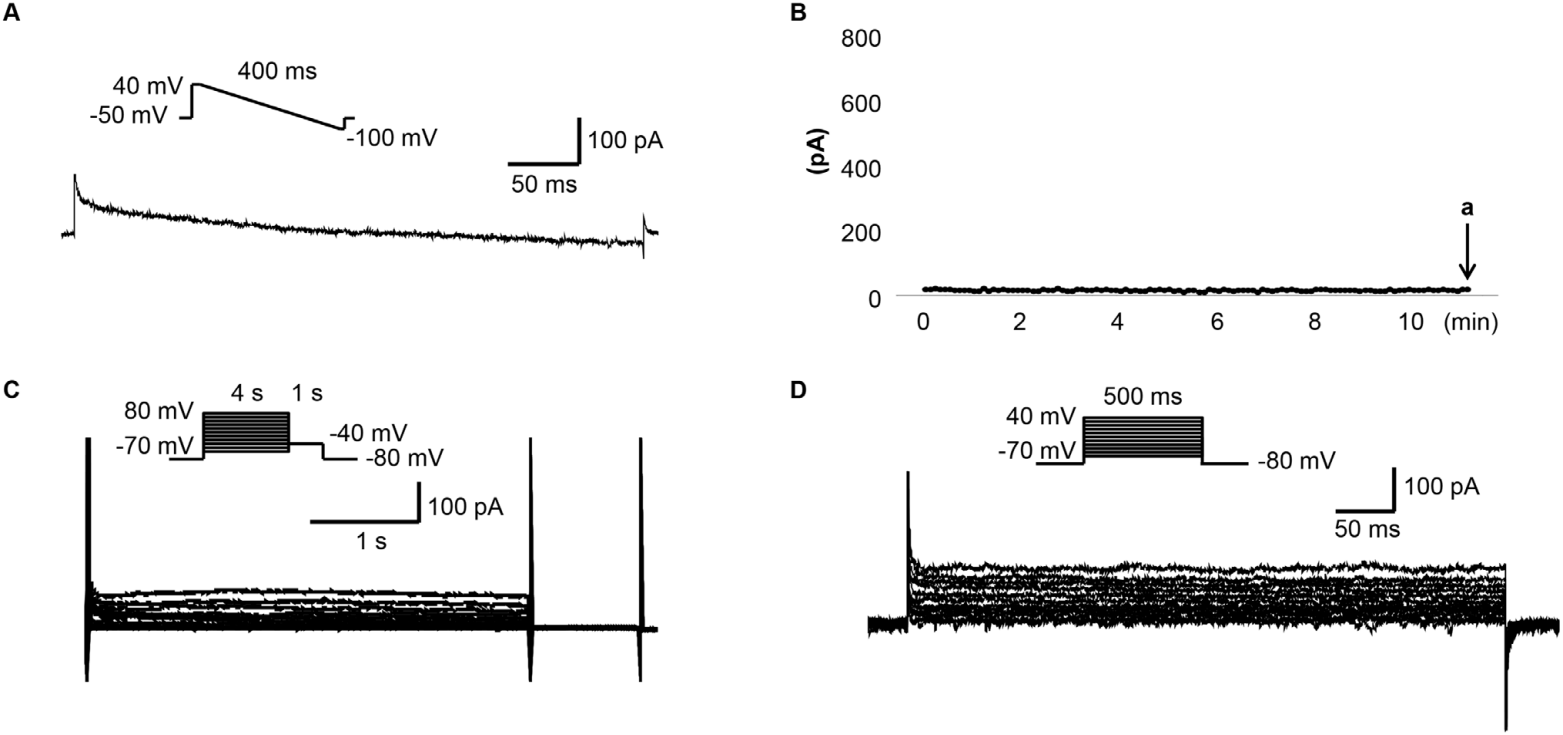
The endogenous K^+^ currents of HEK 293 cells. (A) The representative tracing obtained with ramp protocol shown in the inset at time point indicated by arrow a in (B). The pipette and bath solutions are the same as the ones used in measuring *I*_SK2_. (B) The time course of *I*_SK2_ measured at 0 mV. (C) The representative tracings obtained with the pulse protocol shown in the inset with the pipette and bath solution used in measuring *I*_Ks_. (D) The representative tracings obtained with the pulse protocol shown in the inset with the pipette and bath solutions used in measuring *I*_Kr_, *I*_K1_ and *I*_to_.

**Figure 2:**
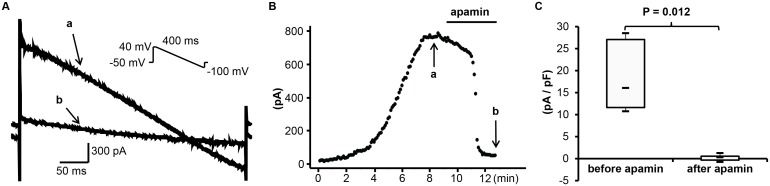
Effect of apamin on *I*_SK2_ in transfected HEK 293 cells. (A) The representative *I*_SK2_ tracings obtained by the descending voltage ramp protocol shown in the inset before (a) and after (b) apamin at time points indicated by arrows a and b in (B). (B) The time course of *I*_SK2_ at 0 mV. (C) The summary of current density before and after apamin.

Figure 1 shows that untransfected HEK 293 cells expressed very low levels of endogenous potassium currents (<100 pA) compared to the nA levels of currents observed after transfection with various ion channels (see figure legends and subsequent figures). Figure 2A and 2B show the representative tracings and time course of *I*_SK2_ in transfected HEK 293 cells, induced by a repetitive voltage-ramp pulses (from +40 to –100 mV, 400 ms duration) from a holding potential of –50 mV. A total 8 cells were tested at 37°C. The currents became stable 4∼8 minutes after the whole-cell configuration was made. Subsequent application of apamin (500 nM) reduced the currents by 99±4%. Figure 2C shows the summary data before and after apamin.

#### Apamin does not inhibit I_Na_

**Figure 3:**
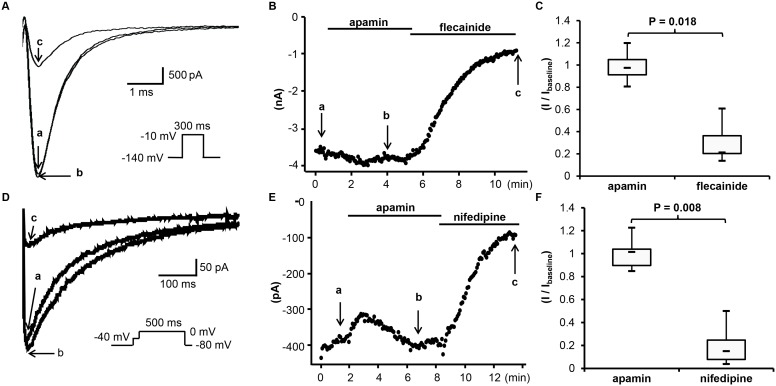
Effects of apamin on *I*_Na_ and *I*_Ba_ in transfected HEK 293 cells. (A) The representative *I*_Na_ tracings obtained by the pulse protocol shown in the inset before apamin (a), after apamin (b) and after flecainide (c) at time points indicated by arrows a through c, respectively, in (B). (B) The time course of peak *I*_Na_ measured at –10 mV. (C) The summary of drug effects normalized to baseline. (D) The representative *I*_Ba_ tracings at 0 mV obtained by the pulse protocol shown in the inset before apamin (a), after apamin (b) and after nifedipine (c) at time points indicated by arrows a through c, respectively, in (E). (E) The time course of peak *I*_Ba_ measured at 0 mV. (F) The summary of drug effects normalized to baseline.

Figures 3A and 3B show the representative tracings and time course of *I*_Na_ at a frequency of 20/min. The *I*_Na_ was induced by a repetitive depolarization pulse (to –10 mV for 300 ms) from a holding potential of –140 mV. All experiments were carried out in room temperature. A total of 9 cells were tested and no significant inhibition or enhancement was observed after adding 500 nM apamin. The median baseline current density was –261 [–282;–145] pA/pF. The averaged current density after apamin was –268 [–379;–132] pA/pF (n = 9, p = 0.767, compared to the baseline). The averaged current density after flecainide was –57 [–70;–47] pA/pF (n = 7; p = 0.018 compared to post apamin, p = 0.018 compared to baseline). Figure 3C shows the summary of drug effects normalized to the baseline.

#### Apamin does not inhibit I_Ba_

Figures 3D and 3E show the representative tracings and time course of *I*_Ba_ in the presence of apamin 500 nM or nifedipine 2 µM. *I*_Ba_ was induced by a step pulse protocol (to 0 mV for 500 ms) from a holding potential of –80 mV and a brief prepulse at –40 mV. A total of 8 cells were tested. No significant effects of apamin were observed on *I*_Ba_. The baseline current density of *I*_Ba_ was –16 [–20;–10] pA/pF. The current density after apamin was 17 [–19;–13] (n = 8, p = 0.953, compared to baseline), and –1.6 [–3.2;–1.3] pA/pF after nifedipine (n = 8; p = 0.008 compared to post apamin, p = 0.008 compared to baseline). *I*_Ca_ had also been studied using 1.8 mM Ca^2+^ in the external solution. However, it was difficult to study the effects of apamin on *I*_Ca_ due to a marked rundown phenomenon. Apamin did not show significant effects during rundown (Figure S1).

#### Apamin does not inhibit I_Ks_

**Figure 4:**
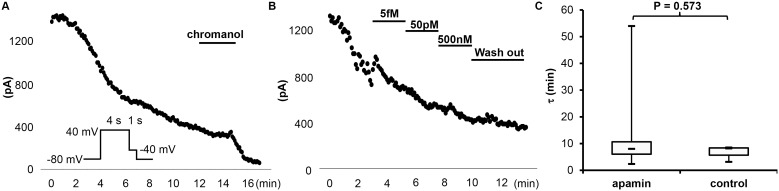
Effects of different concentrations of apamin on the rundown course of *I*_Ks_ in transfected HEK 293 cells. (A) An observation experiment without apamin treatment showing time-dependent rundown, obtained with the pulse protocol shown in the inset with chromanol 293B at the end. (B) The representative time course of *I*_Ks_ treated with different concentrations of apamin. (C) The time constant (τ) of the rundown curve with (n = 10) and without (n = 3) apamin.

**Figure 5:**
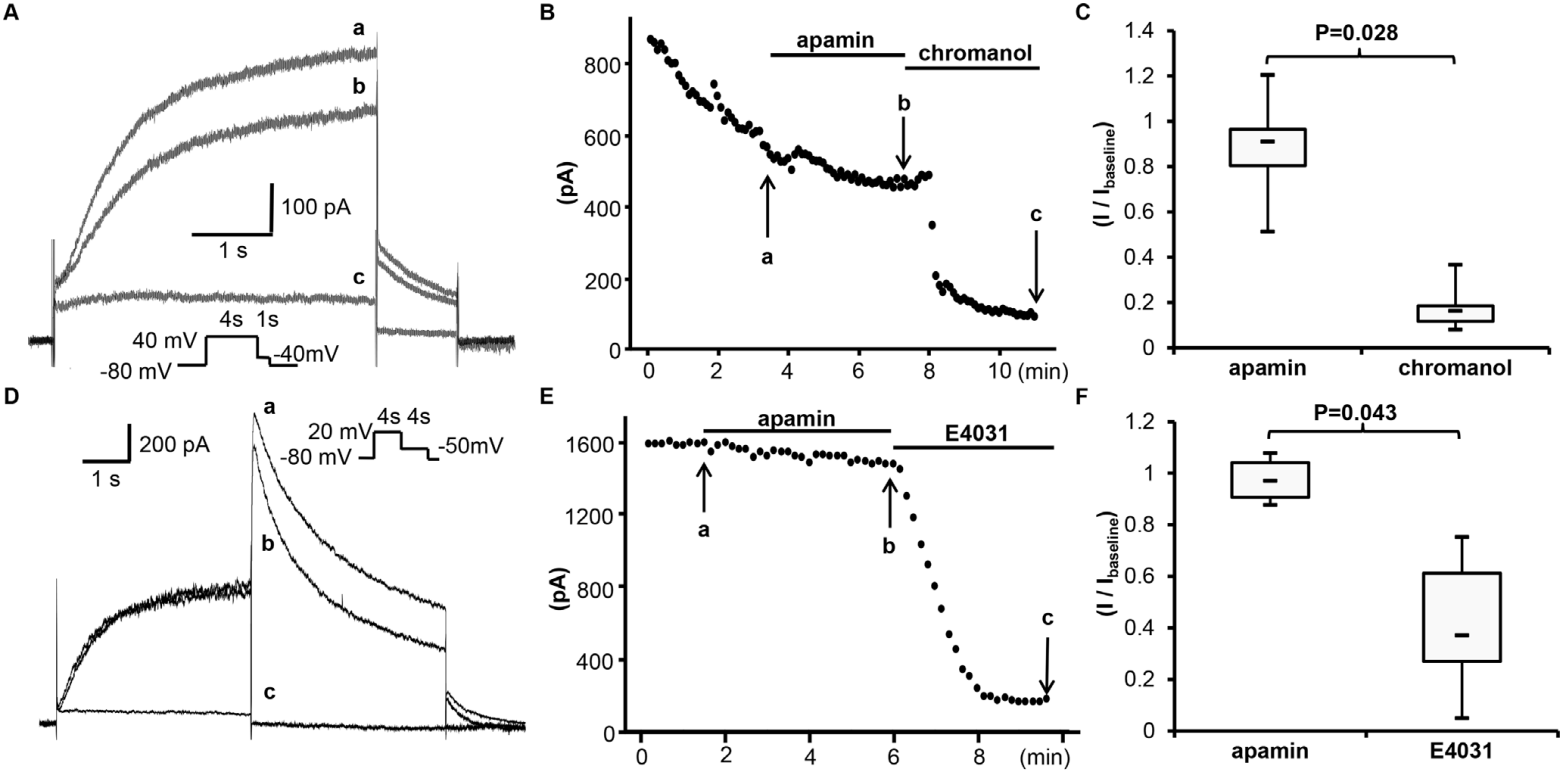
Effects of apamin on *I*_Ks_ and *I*_Kr_ in transfected HEK 293 cells. (A) The representative tracings of *I*_Ks_ obtained by pulse protocol shown in the inset before apamin (a), after apamin (b) and after chromanol (c) at time points indicated by arrows a through c, respectively, in (B). (B) The time course of peak *I*_Ks_ at 40 mV. (C) The summary of drug effects normalized to baseline. (D) The representative tracings of *I*_Kr_ obtained by a pulse protocol shown in the inset before apamin (a), after apamin (b) and after E4031 (c) at time points indicated by arrows a through c in (E). (E) The time course of peak *I*_Kr_ at 20 mV. (F) The summary of drug effects normalized to baseline.

A rundown phenomenon was also observed in the study of *I*_Ks_ (Figure 4A). Various concentrations of apamin (from 0.5 fM to 500 nM) were applied during rundown, but the time course of rundown was not affected (Figure 4B). Figure 4C summarizes the time constant (τ) of rundown with and without apamin. There were no significant differences between the two. Figures 5A and 5B show the representative tracings and time course of *I*_Ks_. *I*_Ks_ was induced with a 4s depolarization pulse protocol (to +40 mV) from a holding potential of –80 mV. The baseline current density of *I*_Ks_ was 28 [20]; [37] pA/pF. After apamin, the average current density was 23 [18]; [32] pA/pF (n = 10, p = 0.037, compared to the baseline). After adding 50 µM Chromanol 293B, the current density was reduced to 4.7 [4.1;6.7] pA/pF (n = 6; p = 0.028 compared to post apamin, p = 0.028 compared to baseline). Figure 5C shows the summary of drug effects normalized to baseline.

#### Apamin does not inhibit I_Kr_

Figures 5D and 5E represent tracings and the time course of apamin effect on *I*_Kr_. The current was induced by a depolarization pulse (to +20 mV for 4 s in duration) from a holding potential of –80 mV, and measured as the peak tail current at –50 mV, repeated every 10 s. Apamin had no significant effect. The baseline current density of *I*_Kr_ was 28 [24]; [30] pA/pF, and was 27 [24]; [29] pA/pF after apamin (n = 6, p = 0.345, compared to baseline). The current density was reduced to 10 [8]; [14] pA/pF by E4031 (n = 5; p = 0.043 compared to post apamin, p = 0.043 compared to baseline). Figure 5F shows the summary of drug effects normalized to baseline.

#### Apamin does not inhibit I_K1_

**Figure 6:**
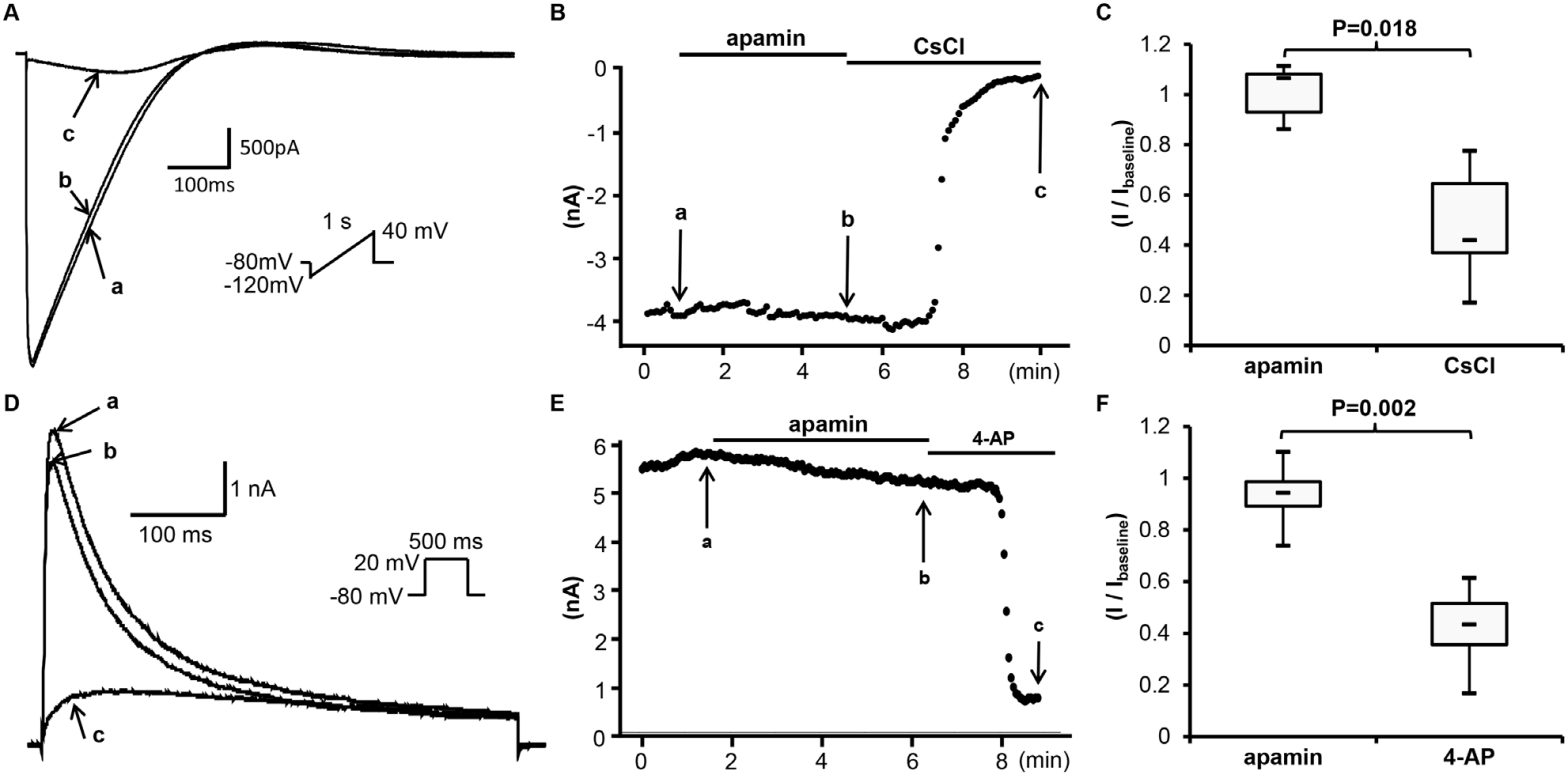
Effects of apamin on *I*_K1_ and *I*_to_ in transfected HEK 293 cells. (A) The representative tracings of *I*_K1_ by ascending voltage ramp protocol shown in the inset before apamin (a), after apamin (b) and after CsCl (c) at time points indicated by arrows a through c, respectively, in (B). (B) The time course of *I*_K1_ at –100 mV. (C) The summary of drug effects normalized to baseline. (D) The representative tracings of *I*_to_ obtained by a pulse protocol shown in the inset before apamin (a), after apamin (b) and after 4-AP (c) at time points indicated by arrows a through c, respectively, in (E). (E) The time course of peak *I*_to_ at 20 mV. (F) The summary of drug effects normalized to baseline.

Figures 6A and 6B show the representative time course and tracings of *I*_K1_ in the absence and presence of apamin (500 nM). The *I*_K1_ was induced by a ramp pulse protocol between –120 mV and 40 mV (1 s in duration, every 5 s) from a holding potential of –80 mV. The current at –100 mV was monitored and shown in Figure 4B. No significant effects were observed after adding apamin. The baseline current density of *I*_K1_ was –46 [–48;–40] pA/pF. After apamin administration, the average current density was –46 [–51;–35] pA/pF (n = 7, p = 0.612, compared to baseline). CsCl reduced the current density to –18 [–27;–15] pA/pF (n = 7; p = 0.018 compared to post apamin, p = 0.018 compared to baseline). Figure 6C shows the summary of drug effects normalized to baseline.

#### Apamin does not inhibit I_to_

Figures 6D and 6E show the effect of apamin on *I*_to_. The current was induced by a repetitive depolarization pulse (+20 mV for 500 ms in duration) from a holding potential of –80 mV. Apamin had no significant effect. The baseline current density of *I*_to_ was 608 [505;748] pA/pF, and was 606 [454;684] pA/pF after apamin (n = 13, p = 0.052, compared to baseline). The current density was reduced to 247 [228;323] pA/pF by 4-aminopyridine (n = 12; p = 0.001 compared to apamin’s effect, p = 0.002 compared to baseline). Figure 6F shows the summary of drug effects normalized to baseline.

### Studies in Rabbit Cardiomyocytes

#### Apamin does not inhibit the native I_Ca_

**Figure 7:**
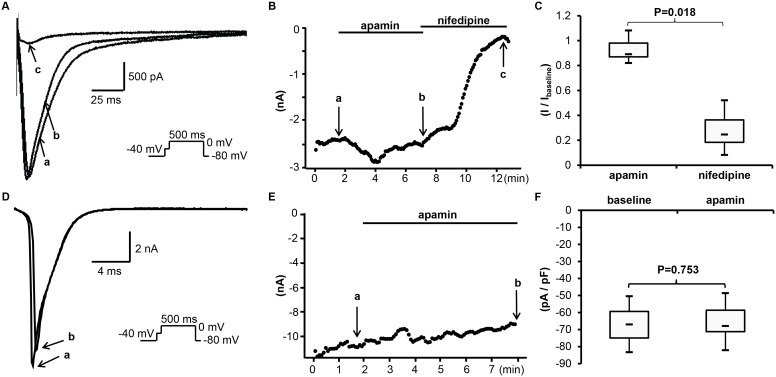
Effects of apamin on *I*_Ca_ and *I*_Na_ in rabbit cardiomyocytes. (A) The representative *I*_Ca_ tracings obtained by a pulse protocol shown in the inset before apamin (a), after apamin (b) and after nifedipine (c) at time points indicated by arrows a through c, respectively, in (B). (B) The time course of peak *I*_Ca_ measured at 0 mV. (C) The summary of drug effects normalized to baseline. (D) The representative tracings of *I*_Na_ obtained by a pulse protocol shown in the inset before apamin (a), after apamin (b) at time points indicated by arrows a and b, respectively, in (E). (E) The time course of peak *I*_Na_ at –40 mV. (F) The summary of current densities before and after apamin.

Figures 7A and 7B show the representative tracings and time course of *I*_Ca_ in the presence of apamin 500 nM or nifedipine 2 µM. *I*_Ca_ was induced by a step pulse protocol (to 0 mV for 500 ms) after a brief prepulse to –40 mV from a holding potential of –80 mV to inactivate *I*_Na_. Apamin had no significant effects on *I*_Ca_. The current density of *I*_Ca_ averaged –16 [–17;–14] pA/pF at baseline, and –14 [–15;–13] pA/pF after apamin (n = 7, p = 0.091, compared to the baseline). After adding 2 µM of nifedipine, the current density was reduced to –3.9 [–5.8;–2.6] pA/pF (n = 7; p = 0.018 compared to post apamin, p = 0.018 compared to baseline). Figure 7C shows the summary of drug effects normalized to baseline.

#### Apamin does not inhibit the native I_Na_

In the same experiments, the native cardiac *I*_Na_ was also measured during the prepulse to –40 mV. Figure 7D and 7E showed the representative tracings and time course of *I*_Na_ before and after apamin. There was no significant change after adding apamin. The baseline current density of *I*_Na_ was –67 [–75;–59] pA/pF, and was –68 [–71;–59] pA/pF after apamin (n = 6; p = 0.753 compared to the baseline). Figure 7F shows the summary of drug effects.

## Discussion

We found that at a concentration of 500 nM, apamin has no significant effects on major cardiac ion currents that underlie the action potential in human hearts, including L-type Ca^2+^, Na^+^ and the major K^+^ currents (*I_Ks_*, *I_Kr_*, *I_K1_*, *I_to_*). This finding suggests that apamin at this concentration can be used to study the role of SK currents in human cardiomyocytes.

### 

#### Apamin as a specific ion channel inhibitor

Apamin is a peptide toxin isolated from Western honey bees [34]. When injected with 0.5 mg/kg or more of apamin, mice develop neurological symptoms including spasms, jerks and convulsions of apparently spinal origin [34]. Subsequent studies showed that apamin is a highly selective SK channel inhibitor in the central nervous system. Because SK channels are the only known targets for apamin, the effects of apamin at the molecular, cellular, and behavioral levels may be ascribed to SK channel blockade [35]. The specificity of apamin in the central nervous system has contributed significantly to the understanding of SK channel function in controlling activation and repolarization of neurons. Since 2003, apamin-sensitive K currents have also been known to be present in cardiac tissues and play an important role in atrial repolarization [5]–[12]. Apamin also prolongs the action potential duration in diseased ventricles, such as in heart failure, myocardial infarction and after atrioventricular block [14]–[16], [18], [19]. However, because previous studies showed that apamin inhibited L-type Ca^2+^ currents [20]–[22] and Na^+^ currents [23] in fetal heart tissue, it is possible that apamin also has non-specific effects on ion channels in adult cardiac tissues. If this is the case, the validity of all research using apamin as a SK inhibitor to explore the role of SK in the heart would in question. For example, if apamin can inhibit any one of the major repolarization currents, such as *I*_Ks_ or *I*_Kr_, then the prolongation of action potential duration after apamin demonstrated in all optical mapping or patch clamp studies may be a result of inhibition of those major ionic currents, and not exclusively from the inhibition of SK currents [15], [36], [37]. If apamin inhibits *I*_to_, then the observed effect of apamin in atria may be explained by *I*_to_ inhibition since that current is abundantly present in the atria [38]–[41]. If apamin could affect *I*_K1_, then the change of arrhythmia burden after apamin administration could in part come from resting membrane potential shift due to *I*_K1_ inhibition [17], [42], [43]. If apamin could affect *I*_Na_, then apamin would affect propagation velocity and excitability of heart tissue and thereby influence the arrhythmogenesis. In addition, if apamin inhibits *I*_CaL_, then the latter effect may explain the flattening of action potential duration restitution curve in failing ventricles by apamin [15]. Therefore, if apamin is a nonspecific ion channel blocker, the effects of apamin on arrhythmogenesis may not come from SK channel inhibition alone. Because an extensive literature search showed no other studies that have tested the specificity of apamin in human cardiac ion channels, our study is both novel and important for interpreting the antiarrhythmic and proarrhythmic mechanisms of SK current inhibition evaluated using apamin.

In the present study, we used HEK 293 cell line and isolated rabbit ventricular myocytes to study apamin specificity. The HEK 293 cell line was originally derived from human embryonic kidney cells and has the advantage of high transfectability and being easy to culture. This cell line has relatively small endogenous currents compared to the currents expressed in transfected cells (Figure 1), making contamination by endogenous currents insignificant. HEK 293 cells have been widely used to express cloned cardiac ion channels, including Nav1.5 [44], [45], Cav1.2 [46]–[48], Kv7.1 [49], Kv11.1 [31], [50], Kir2.1 [51] and Kv4.3 [52] channels. The currents exhibited in the present experiments are consistent with those reports. The concentration of apamin tested most commonly in this study was 500 nM, which is more than 1000 times the reported IC_50_ (0.027∼0.095 nM) of the SK2 currents in HEK 293 cells [53]–[55]. Five hundred nM is also higher than the dose used to block Ca^2+^ and Na^+^ currents in chick embryo reaggregates by Bkaily et al [20], [21], [23]. In addition to HEK 293 cells, we also performed studies in isolated rabbit ventricular myocytes and showed that apamin failed to block either *I*_Na_ or *I*_CaL_. The differences between our results and those reported by Bkaily et al. might have come from species differences or the differences of isoforms between adult and fetal ion channels.

#### Limitations of the study

Because we did not test the fetal isoform-encoded ionic currents or ionic currents of various possible splicing isoforms in all animal species, our results are only applicable to the most common isoforms of adult human cardiac cells. It is also possible that in native cardiac myocytes, some of these channels have different subunit combinations that we did not test, or their regulation may be different. In the intact heart, ionic currents are also affected by autonomic nerves sensitive to apamin. Since cell environments of HEK cells and rabbit cardiomyocytes are very different from human cardiomyocytes, there is a possibility that apamin may show some effects on the ion channels that we studied in human cardiomyocytes. Further studies using human cells will be warranted.

## Conclusions

We conclude that apamin does not have significant effects on the most common isoforms underlying the major human cardiac ion channels. These findings support prior evidence that apamin is a highly selective inhibitor of SK current in the cardiomyocytes.

## Supporting Information

Figure S1 **A representative time course of** ***I*** **_Ca_ in transfected HEK 293 cells measured at 0 mV.** Apamin and nifedipine was added during rundown.
(TIF)
