# Correction: Apamin Does Not Inhibit Human Cardiac Na^+^ Current, L-type Ca^2+^ Current or Other Major K^+^ Currents

**DOI:** 10.1371/journal.pone.0104445

**Published:** 2014-07-30

**Authors:** The PLOS ONE Staff

There are errors in the published article. In the Results, and in the Abstract, all numbers appearing in square parentheses are data numbers, not reference numbers, and should not be hyperlinked to references.

There are errors in Figures 1 and 4. The y-axis is missing from the graphs in Figure 1(B) and in Figure 4(A) and (B). Please view the correct figures below.

**Figure 1:**
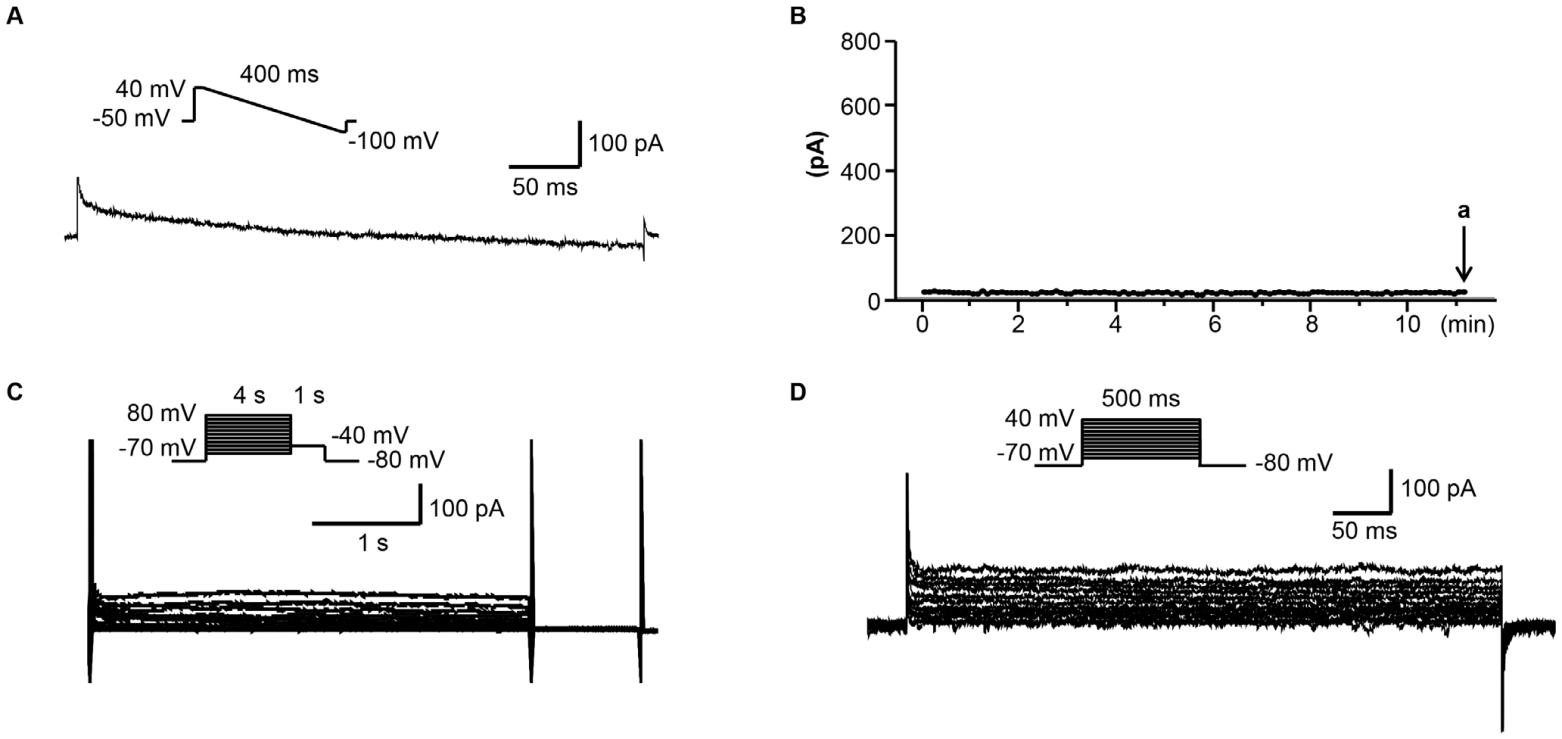
The endogenous K^+^ currents of HEK 293 cells. (A) The representative tracing obtained with ramp protocol shown in the inset at time point indicated by arrow a in (B). The pipette and bath solutions are the same as the ones used in measuring *I*_SK2_. (B) The time course of *I*_SK2_ measured at 0 mV. (C) The representative tracings obtained with the pulse protocol shown in the inset with the pipette and bath solution used in measuring *I*_Ks_. (D) The representative tracings obtained with the pulse protocol shown in the inset with the pipette and bath solutions used in measuring *I*_Kr_, *I*_K1_ and *I*_to_.

**Figure 4:**
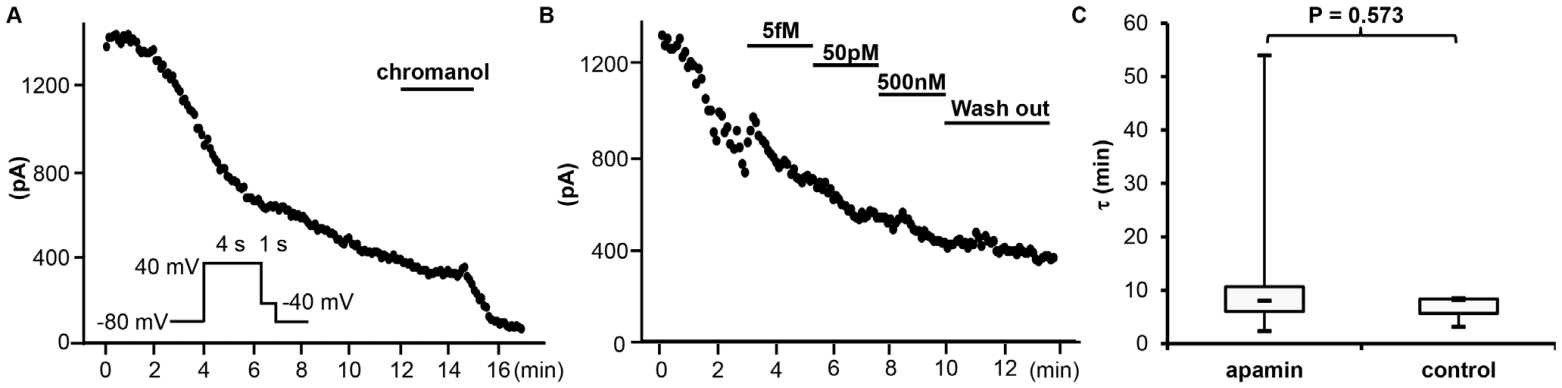
Effects of different concentrations of apamin on the rundown course of *I*_Ks_ in transfected HEK 293 cells. (A) An observation experiment without apamin treatment showing time-dependent rundown, obtained with the pulse protocol shown in the inset with chromanol 293B at the end. (B) The representative time course of *I*_Ks_ treated with different concentrations of apamin. (C) The time constant (τ) of the rundown curve with (n  =  10) and without (n  =  3) apamin.
